# Biophysical Mechanism for Ras-Nanocluster Formation and Signaling in Plasma Membrane

**DOI:** 10.1371/journal.pone.0006148

**Published:** 2009-07-09

**Authors:** Thomas Gurry, Ozan Kahramanoğulları, Robert G. Endres

**Affiliations:** 1 Centre for Integrated Systems Biology at Imperial College, Imperial College London, London, United Kingdom; 2 Department of Applied Mathematics and Theoretical Physics, University of Cambridge, Cambridge, United Kingdom; 3 Department of Computing, Imperial College London, London, United Kingdom; 4 Division of Molecular Biosciences, Imperial College London, London, United Kingdom; Keio University, Japan

## Abstract

Ras GTPases are lipid-anchored G proteins, which play a fundamental role in cell signaling processes. Electron micrographs of immunogold-labeled Ras have shown that membrane-bound Ras molecules segregate into nanocluster domains. Several models have been developed in attempts to obtain quantitative descriptions of nanocluster formation, but all have relied on assumptions such as a constant, expression-level independent ratio of Ras in clusters to Ras monomers (cluster/monomer ratio). However, this assumption is inconsistent with the law of mass action. Here, we present a biophysical model of Ras clustering based on short-range attraction and long-range repulsion between Ras molecules in the membrane. To test this model, we performed Monte Carlo simulations and compared statistical clustering properties with experimental data. We find that we can recover the experimentally-observed clustering across a range of Ras expression levels, without assuming a constant cluster/monomer ratio or the existence of lipid rafts. In addition, our model makes predictions about the signaling properties of Ras nanoclusters in support of the idea that Ras nanoclusters act as an analog-digital-analog converter for high fidelity signaling.

## Introduction

Plasma membrane heterogeneity is a key concept in molecular cell biology due to its role in protein sorting and specificity of signaling [1]–[3]. Although the diversity of the membrane's lipid components is partly responsible for this heterogeneity [4], the role played by membrane proteins is less well understood. Members of the Ras protein superfamily [5], [6] have been observed to form dynamic, non-overlapping domains called nanoclusters in the inner leaflet of the plasma membrane [7]–[10]. While the lateral segregation of Ras may provide evidence towards the existence of small, dynamic rafts [11], the definition and even existence of rafts remains disputed [12]. In addition to its connection to the lipid-raft concept, Ras has attracted immense interest due to its fundamental role in a multitude of cellular processes, including cell proliferation, survival, and motility. Most importantly, Ras genes are found to be mutated in 30% of human cancers [13]–[15], making their products extremely important therapeutic targets [16]. While the intracellular biochemistry of Ras genes is well documented, the biophysical mechanism and role of Ras clustering in the plasma membrane remains little understood.

Ras GTPases are small (21 kDa), lipid-anchored peripheral membrane proteins involved in signal transduction [13]. Three Ras isoforms H-Ras, K-Ras and N-Ras are expressed in all mammalian cells. These isoforms contain a conserved G-domain which binds guanine nucleotides [17]. Ras effectively acts as a molecular switch for the signal, with “on” (GTP-bound) and “off” (GDP-bound) states, the former promoting an association with and activation of effector proteins. Although nearly identical with respect to their catalytic and effector-binding properties, H-Ras, N-Ras and K-Ras have very different biological roles. This functional distinction is believed to result at least in part from the differential membrane compartmentalization of Ras isoforms [18], [19]. The different distribution of Ras proteins in cellular membranes dictates unique spatio-temporal patterns of activation of effector pathways. A classical example of a pathway involving Ras is the Ras-Raf-MEK-ERK pathway, a mitogen-activated protein kinase (MAPK) cascade involved in cell proliferation, differentiation, and apoptosis. In this pathway, the epidermal growth factor receptor (EGFR), a receptor tyrosine kinase, is stimulated. This leads to recruitment and activation of guanine nucleotide exchange factors (GEFs), which, by interacting with the Ras G-domains, promote the exchange of GDP for GTP [17] and lead to Ras activation. Ras  GTP activates protein kinase Raf and initiates the phosphorylation cascade, ultimately leading to double phosphorylated ERK (ERKpp), which then travels into the nucleus and phosphorylates transcription factors [20]. Among other purposes, such cascades can lead to a massive amplification of the original signal [20].

Experimental evidence for the formation of nanoclusters (termed clusters from now on) is provided by *in vivo* and *in vitro* experiments. Fluorescence resonance energy transfer (FRET) studies show that activation by EGF leads to significant decrease in Ras lateral diffusion, suggesting the existence of Ras  GTP clusters [21]. A very similar result was obtained by single-molecule fluorescence microscopy, where GTP-binding of Ras leads to slowly diffusing active Ras molecules [22]. Single particle tracking (SPT) studies of fluorescently labeled Ras have also demonstrated transient immobility of Ras (lasting less than 1 s) with high temporal resolution, interspersed with periods of free Brownian motion [23]. Furthermore, spatial statistics of fluorescently labeled Raf have shown that Ras and Raf cluster together [24]. It is therefore believed that active Ras forms signaling platforms, which recruit and activate Raf. As signaling platforms are Ras-isoform specific, the signal diversity observed between H-Ras, K-Ras and N-Ras is in part the result of differential clustering properties in these isoforms [7].

**Figure 1:**
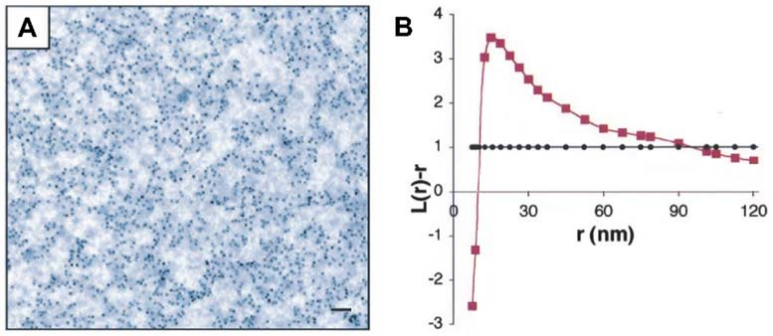
Experimental immunoEM data and statistical clustering analysis. (A) Electron micrograph of immunogold-labeled Ras domain (GFP-tH where tH is minimal plasma membrane targeting motifs of H-Ras) in an *in vitro* plasma membrane sheet. Scale bar is 100 nm. (B) Corresponding point-pattern analysis (red) and 99% confidence interval (black). ©Prior *et al.* (2003), originally published in *The Journal of Cell Biology*. doi:10.1083/jcb.200209091 [7].

Direct evidence for protein clustering in a membrane can be obtained from high-resolution electron microscopy (EM). However, Ras is too small and not electron dense enough to be observed directly. To circumvent this problem, Prior *et al.* used GFP-Ras fusion constructs, which were treated with gold-labeled anti-GFP antibodies. The resulting immunogold point patterns were visualized with EM (immunoEM) to quantitatively describe Ras clustering (Fig. 1) [7]. It was found that the classical raft model, wherein a fixed number of lipid rafts accommodate a fixed fraction of raft-inserted proteins, is incompatible with the observed gold point patterns [11]. Indeed, for increasing expression levels, the classical model predicts an increase in the number of proteins per raft, and therefore a greater degree of clustering. To describe the data, Plowman *et al.* developed an alternative raft model, in which the size of Ras clusters remains constant. Assuming a constant, expression level-independent ratio of Ras in clusters to Ras monomers (cluster/monomer ratio) results in the formation of more rafts as expression increases, and supports the notion that lipidated molecules such as Ras can drive the formation of rafts in order to create signaling platforms [11]. This alternative model predicts that 40% of active Ras molecules form clusters of radius 6–12 nm, each containing about seven Ras molecules, and 60% are randomly distributed monomers [24]. While simulations of this model fit immunoEM data, they do not provide a biophysical explanation for Ras clustering. Furthermore, these simulations violate laws of equilibrium physics. Specifically, the law of mass action predicts an increase in the fraction of clustered molecules as the expression level is increased (until membrane saturates) [11]. This violation is troublesome as the experiments are done on *in vitro* membrane sheets, where no active, energy-driven processes can limit cluster size. Membrane sheets were fixed (and proteins immobilized) after membrane removal from cells [7], leading to equilibration of membrane and proteins prior to imaging.

Recent experiments even go further and probe the design principles of signaling by Ras clusters. Such studies suggest that, in the Ras-Raf-MEK-ERK pathway, Ras clusters act as an analog-digital-analog converter, where analog, continuous EGF input is converted into digital, fully active clusters. The number of fully active Ras clusters, not the activity of individual Ras molecules, translates into analog ERKpp output [25], [26]. Specifically, these experiments show that Ras mutants with wide-ranging activities lead to the same total cellular ERKpp output [27]. This suggests that Ras clusters act as digital nanoswitches, which become fully activated even for small inputs. Furthermore, the concentrations of active Ras and ERKpp are directly proportional to EGF input [24]. Hence, analog inputs produce analog outputs, mediated by digital Ras clusters.

**Figure 2:**
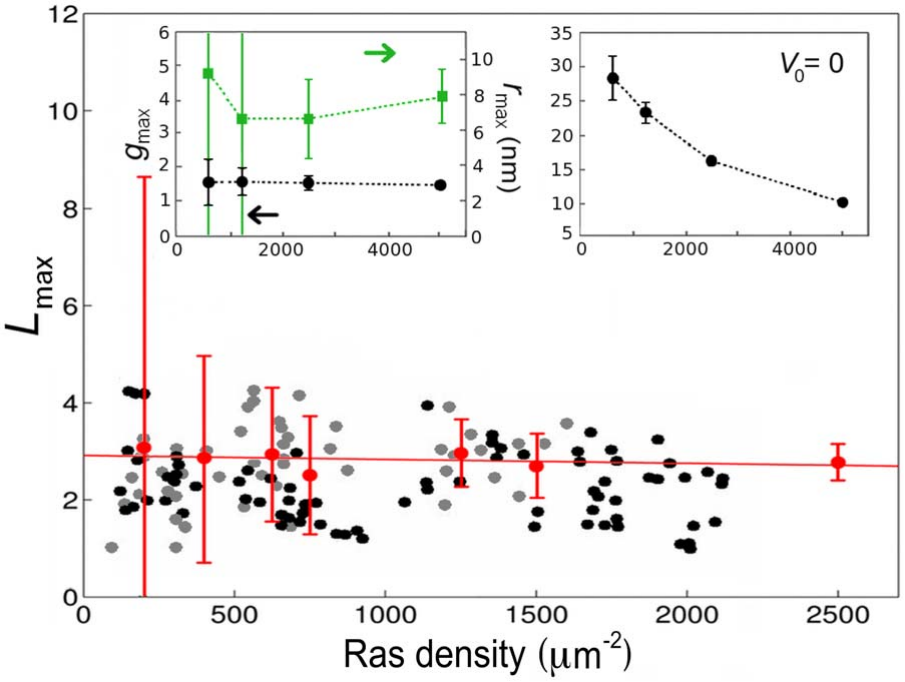
Relation between 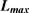 and Ras density *λ* for immunoEM data of gold labeled GFP-tH (black symbols) and RFP-tH (gray symbols), simulation averages and 99% confidence intervals (red), as well as a linear least-squares fit to simulation averages (red line). 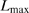
 data points were extracted from Ref. [11] with IMAGE J. (Left inset) 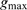 (black) and 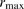 (green) as a function of *λ*.(Right inset) 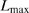 as a function of *λ* without long-range repulsion (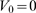). Error bars represent standard deviations. For simulation details, including calculation of confidence intervals, see *Methods*.

Here, we consider a physically-motivated model to study Ras clustering. The model mainly depends on a close-contact, attractive interaction between active Ras molecules (short range 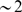 nm) and a repulsive interaction between Ras molecules irrespective of activity (long range 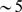 nm). The short-range attraction promotes clustering of active Ras, while the long-range repulsion limits cluster size. Contrarily to previous models, we make no assumption about a constant, expression-level independent cluster/monomer ratio or the existence of lipid rafts, thus circumventing controversy surrounding their actuality. We equilibrate a discretized lattice membrane, occupied with active and inactive Ras molecules, using Monte Carlo simulations. After gold-labeling of Ras molecules from simulation outputs, we perform a statistical clustering analysis. The obtained statistical properties of Ras molecules quantitatively agree with the statistical properties of immuno-gold point patterns for wide-ranging Ras expression levels (Fig. 2) [11]. Our model makes predictions about the signaling properties of Ras clusters, supporting the notion that Ras clusters indeed act as an analog-digital-analog converter [24].

## Results

**Figure 3:**
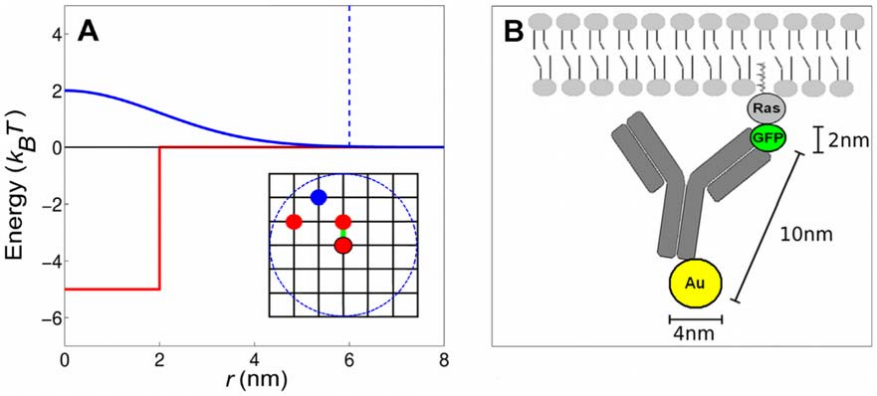
Model ingredients. (A) Short-range attraction (red) and long-range repulsion (blue) as a function of distance between two Ras molecules for the parameters given in *Methods*. Also shown is the cut-off beyond which the repulsive energy is set to zero (blue dashed line). (Inset) Representative part of lattice membrane showing three active Ras molecules (red) and one inactive Ras molecule (blue). Neighboring active Ras molecules interact via the attractive short-range interaction (green bar). The cut-off used for the long-range repulsion is representatively shown for the central Ras (blue dashed circle). (B) Schematic of a gold-labeled antibody associated with a GFP-Ras molecule in the inner leaflet of the plasma membrane.

Prior *et al.* [7] studied Ras clustering in plasma membrane sheets using immunoEM of gold-labeled Ras molecules (Fig. 1A). Gold point patterns were analyzed based on Ripley's *K* function. Specifically, the non-linear transformation 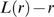 was applied, where *r* is the distance between gold particles. This function is zero for complete randomness, positive for clustering, and negative for depletion (Fig. 1B). Plowman *et al.* [11] used the function's maximal value, termed 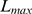 for short, as summary statistics for clustering, and found that 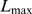 is independent of Ras expression level (Fig. 2, symbols). This was rationalized by an ad hoc clustering model, assuming a constant cluster/monomer ratio. Analysis of immuno-gold patterns is consistent with small clusters, containing approximately 6 to 8 molecules. Here, we use a biophysical model of Ras clustering in the plasma membrane. In our model, a Ras molecule can be in either an active (on) or an inactive (off) state, corresponding to the respective GTP-bound and GDP-bound molecules for wild-type Ras. Both active and inactive Ras are associated with membrane in line with experimental observation [28]. The equilibrium probability of a single Ras molecule to be active depends on the effective free-energy difference between the on and off states, which in turn depends on input signals. We assume that active Ras molecules experience a short-range attraction, driving cluster formation of active Ras, whereas a long-range repulsion limits cluster size (Fig. 3A, main panel). Such a long-range interaction may result from lipid-anchor induced membrane deformations. To obtain equilibrium properties, we approximate the membrane by a square lattice, populated by Ras molecules of a specified density (Fig. 3A, inset), and perform Monte Carlo simulations. For comparison with immunoEM experiments, we added 10nm-long gold-labeled antibodies (maximally one per Ras) to the Ras molecules in the experimentally observed capture ratio (Fig. 3B). We mainly use the four Ras densities given in Table 1. To specifically compare with experiments on varying Ras-expression level (symbols in Fig. 2, main panel), we calculate the 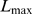 value for additional Ras densities. Note that these experiments are based on the lipid anchor of H-Ras (tH), which has similar clustering properties as active H-Ras [11]. For details on the experiments and our approach, see *Methods*.

**Table 1:** Representative Ras densities with corresponding numbers of Ras molecules on discretized lattice membrane as well as gold densities.

Ras density	Ras per lattice	Gold density
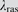 ( 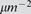 )		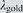 ( 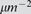 )
625	225	264
1,250	450	525
2,500	900	1,050
5,000	1,800	2,100

**Figure 4:**
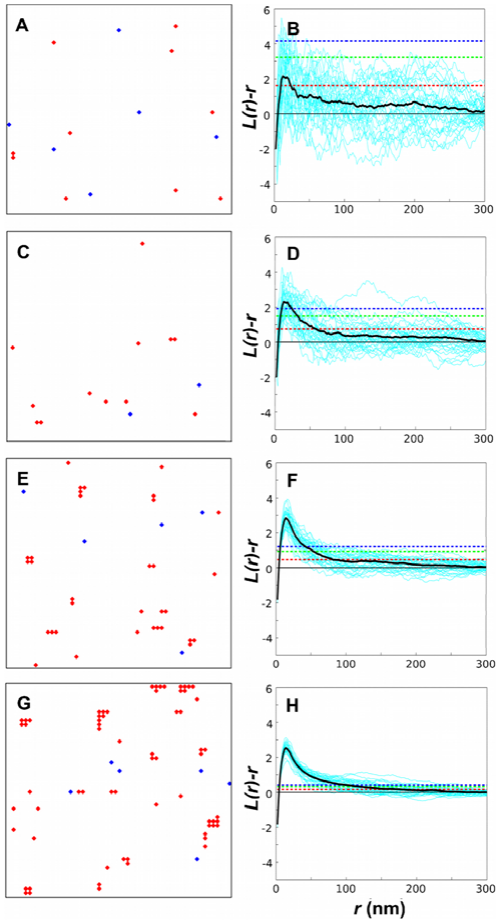
Monte Carlo simulations and point-pattern analysis. Snapshots of equilibrated Ras molecules on lattice membrane (left column; active Ras in red and inactive Ras in blue) and corresponding 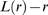 plots (right column) after gold labeling for the four densities from Table 1 (density of Ras molecules increases from top to bottom). Shown in the 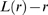 plots are individual simulations (cyan curves), their averages (thick black curves), as well as 68.3%, 95.4%, and 99.0% confidence intervals (red, green, and blue dashed lines, respectively). For simulation details, including calculation of confidence intervals, see *Methods*.

**Figure 5:**
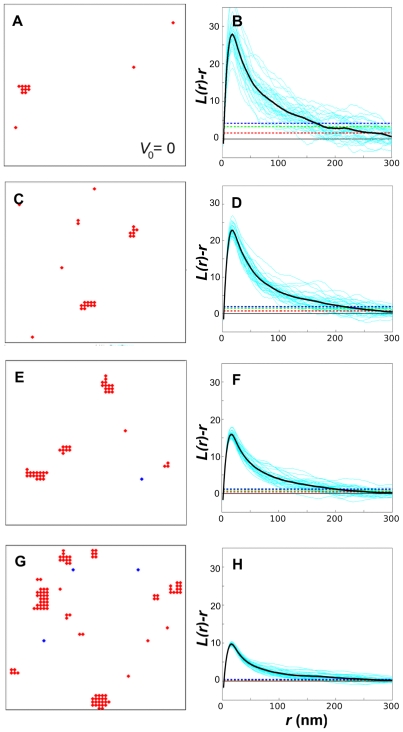
Monte Carlo simulations and point-pattern analysis for conventional clustering model without long-range repulsion (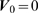). For a description of symbols and lines, see Fig. 4.

**Figure 6:**
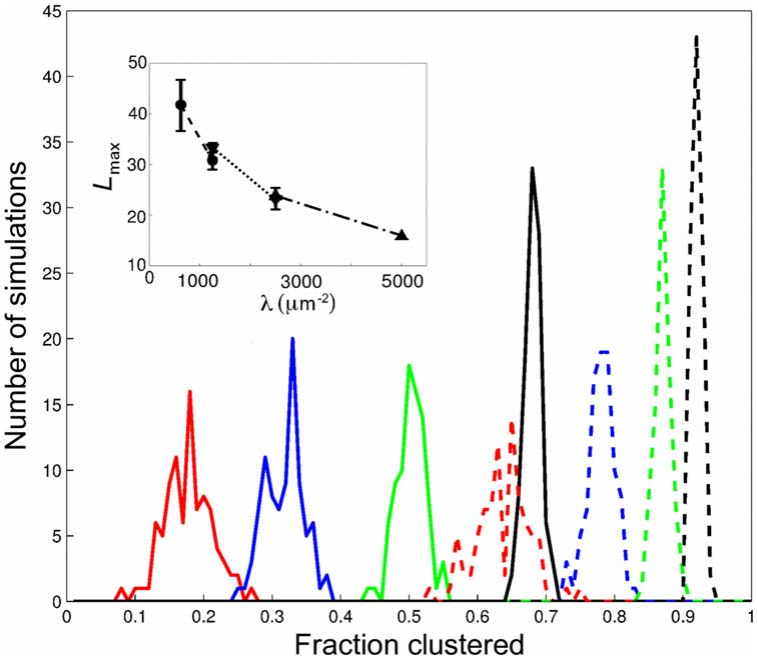
Distributions of Ras fractions in clusters. Different colors correspond to the four Ras densities from Table 1, i.e. 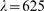 (red), 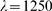 (blue), 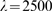 (green), 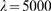 (black) in units of 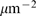. Shown are results with (solid lines) and without (dashed lines) long-range repulsion. A Ras cluster is defined as two or more connected Ras molecules. (Inset) 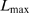 for pairwise constant fractions (overlapping fractions), *i.e.* fraction range 0.72–0.75 for 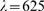 and 1250 (circles and dashed line), fraction range 0.81–0.84 for 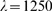 and 2500 (triangles up and dotted line), and fraction range 0.88–0.91 for 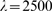 and 5000 (triangles down and dashed-dotted line).

Shown are the four Ras densities used in Figs. 4, 5, and 6. For lattice parameters, see *Methods*.

Figure 4 shows typical, equilibrated membrane lattices for the four Ras densities (left panels) with the corresponding plots of 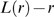 (right panels). For the lowest density, individual 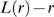 plots are highly variable. To produce meaningful statements about clustering we also show the averaged plot, as well as provide confidence intervals. In line with experiment on varying Ras-expression level, we observe that for our model, 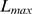 is approximately independent of Ras density (Fig. 2, main panel). The same is true if the analysis is done directly on Ras molecules instead of the gold particles, demonstrating the robustness of the result with respect to the details of Ras labeling by gold. Distance 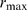, defined as the distance corresponding to 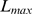, is about 8 nm (Fig. 2, left inset), or equivalently, 4 Ras molecules. Hence, clusters contain few, about 4 to 10, Ras molecules. An alternative clustering analysis based on the pair-correlation function 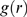 gives similar results, *i.e.* 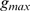 values are independent of *λ* (Fig. 2, left inset). Hence, cluster sizes and their dependence on expression level are in good agreement with previous estimates [11].

We also explored a more conventional clustering model without the long-range repulsion, but maintaining the short-range attraction. As shown in Fig. 5 (left panels), Ras molecules form increasingly larger clusters at increasing Ras densities. Examination of the 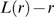 plots (right panels) shows that 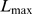 decreases for increasing Ras densities (Fig. 2, right inset), which is in stark contrast to experiments. Hence, limiting the cluster size by the long-range repulsion is a necessary ingredient to correctly describe immunoEM data and, hence, Ras clustering.

Next, we examined the fraction of Ras molecules in clusters. Previous models assumed that the fraction is constant, *i.e.* independent of Ras density. In contrast, Fig. 6 shows that in our model the distribution of the fraction clustered increases significantly with density, indicating that a constant cluster/monomer ratio is not required to describe the immunoEM data in Fig. 2. Also shown in the Fig. 6 is the fraction clustered for the conventional clustering model without the long-range repulsion. The distribution also shifts to higher values with density, although to a lesser extent as fractions are much higher to start out with due to the missing long-range range repulsion. To clearly rule out the conventional clustering model as a suitable model, we tested whether assuming a constant fraction clustered (or equivalently, a constant cluster/monomer ratio) can explain the immunoEM data. For this purpose we collected simulations from different densities but same fraction clustered and compared their 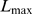 values. However, even with this strong selectivity of simulations, 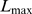 values continued to decrease with increasing density (Fig. 6, inset).

**Figure 7:**
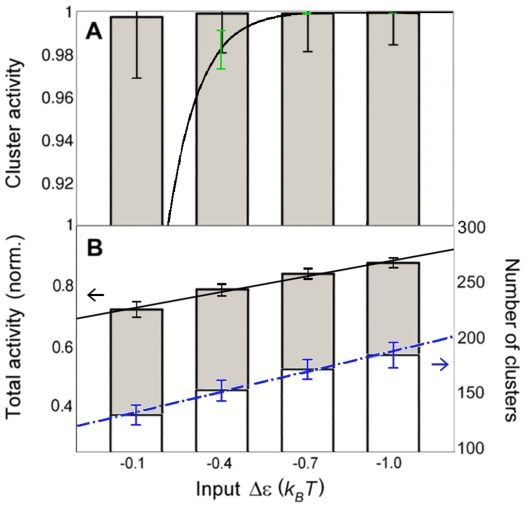
Signaling properties of Ras clusters. (A) Cluster activity as a function of input (parameter 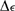). Cluster activity is defined as fraction of active Ras in clusters from simulations (bar chart), where a cluster contains two or more contacting Ras molecules. Also shown is approximate cluster activity 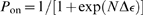, which assumes that all *N* Ras molecules in a cluster (here chose 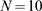) are tightly coupled and hence are either all on (active) or all off (inactive) together (black line). Black error bars show standard deviation and represent intrinsic noise. Green error bars represent extrinsic noise, calculated with noise propagation formula 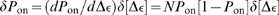 for 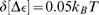. (B) Total activity of all Ras molecules in the membrane, normalized by the total number of Ras molecules (grey bar chart, left axis), and number of Ras clusters (white bar chart, right axis). Also shown are linear fits. Error bars represent standard deviations. To enlarge black error bars for better visualization in *B*, we used the square-root of the total variance from pooled simulations of inputs 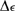, 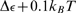, and 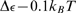.

**Figure 8:**
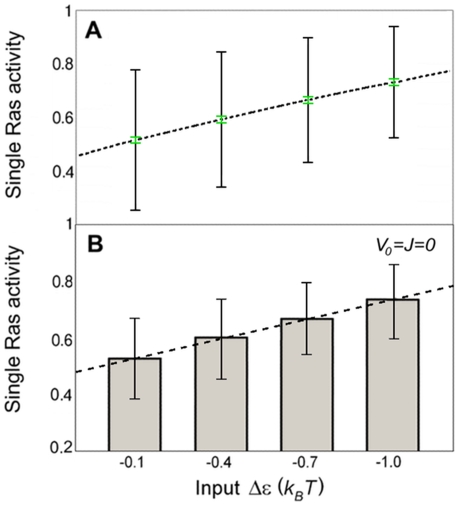
Signaling properties of non-interacting Ras molecules. (A) Activity of single Ras molecule (dashed line; calculated with Eq. 1 for 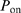) as a function of input (parameter 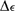). Black error bars represent intrinsic noise, calculated from the square-root of the binomial variance 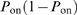. Green error bars are approximately 0.01 in magnitude and represent extrinsic noise, calculated with the noise propagation formula 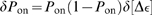 and 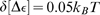. (B) Total activity of all Ras molecules in the membrane, normalized by the total number of Ras molecules (bar chart) and linear fit (dashed line). Error bars represent standard deviation, calculated from the square-root of the total variance from pooled simulations of inputs 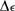, 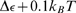, and 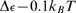.

Figure 7 shows the signaling characteristics of Ras clusters for four different inputs. For input we use the free-energy difference between on (active) and off (inactive) Ras states ( *cf.* Eq. 1). To test if our model produces digital-like nanoswitches, which are fully active even for small inputs, we identified clusters of two or more connected Ras molecules and calculated the cluster activity, *i.e.* the fraction of active Ras molecules in clusters. The bar chart in Fig. 7A shows that our model indeed produces nanoswitches, which are fully active even for small stimuli, as indicated by experiments [27]. In contrast, the activity of a single Ras molecule does not behave like a switch (Fig. 8A, dashed line). Furthermore, Fig. 7B provides the total activity of all Ras molecules in the membrane irrespective of whether Ras molecules belong to clusters or not. We find the total activity is approximately proportional to the input (black line) in the range considered here in line with experiment [24]. In our model, this is due to the fact that the number of Ras clusters is proportional to the input (Fig. 7B, blue line).

There has recently been immense interest in understanding the effect of noise in signal transduction [29], [30]. Biochemical reactions are inherently noisy as they are based on random collisions of molecules. This intrinsic noise is further enhanced by the small number of molecules involved. Furthermore, rate constants may fluctuate, as they depend on external conditions such as other molecules not explicitly considered as part of the biochemical reactions. This extrinsic noise also includes fluctuations in the input itself. To address how Ras signaling is affected by noise, we compare signaling by Ras clusters (Fig. 7) with signaling by non-interacting Ras molecules without clustering ability (Fig. 8). Intrinsic noise is inherently part of our simulations as Ras is allowed to randomly switch between the active and the inactive state. The intrinsic noise for the activity of Ras in clusters (Fig. 7A, black error bars) is significantly less than for the activity of a single Ras molecule (Fig. 8A, black error bars) since clusters are fully active and hence suppress random switching. This difference in intrinsic noise is reduced when considering the intrinsic noise of the total activity from all Ras molecules in the membrane, which is only slightly smaller for Ras clusters (Fig. 7B, black error bars) than for non-interacting (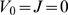) Ras molecules (Fig. 8B, black error bars). This is due to the fact that the number of Ras clusters, which is necessarily smaller than the number of Ras molecules, can fluctuate significantly (Fig. 7B, blue error bars). Most importantly, Ras clusters are more robust to input noise, at least for sufficiently large inputs, than non-interacting Ras molecules (by comparison of green error bars in Fig. 7A and Fig. 8A). Here, input noise represents fluctuations in input much faster than assembly/disassembly of clusters but slower than Ras signaling. Therefore, it is assumed that extrinsic noise only affects the activity of Ras molecules, not clustering itself (see captions of Fig. 7A and Fig. 8A for details). This shows that Ras clusters have superior signaling properties compared to non-interacting Ras molecules without clustering ability.

## Discussion

Different Ras isoforms are known to form nonoverlapping signaling clusters [8], [18], important for localized signaling of the Ras-Raf-MEK-ERK pathway [31], [32], involved in cell proliferation, differentiation, and apoptosis [20]. In addition to the fundamental importance of Ras in this pathway, Ras mutations are found in 30% of human cancers [13]–[15]. Ras clusters are also considered evidence of lipid rafts [11]. Lipid rafts have attracted considerable interest due to their alleged role in protein sorting and specificity of signaling [1]–[3]. In this work, we provided a biophysical model of Ras clustering, and compared results with gold-point patterns obtained from immunoEM of plasma membrane extracts (Fig. 1). In particular, we obtained that clustering of Ras molecules, *i.e.* the cluster/monomer ratio, is independent of expression level (Fig. 2), in line with experiments on the lipid anchor of H-Ras (tH) [11]. In our model, as well as in experiments, clustering is quantified by the maximum value (termed 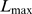) of function 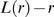 [11], where *r* is the distance between gold particles. Our model has two main ingredients exemplified in Fig. 3: (1) a short-range attraction between active Ras molecules ( *e.g.* Ras  GTP) promoting clustering, and (2) a long-range repulsion between Ras molecules, which limits cluster size.

Another important feature, which makes our model fundamentally different from previous Ras clustering models [11], [24], is that the fraction of clustered Ras molecules is not a model parameter [11] but a prediction from our simulations. Indeed, if we calculate the fraction of clustered molecules for the four densities from Table 1, we obtain the distributions shown in Fig. 6. The fraction of clustered Ras increases with density, indicating that the assumption of a constant cluster/monomer ratio [11], [24] is misleading for describing the immunoEM data for different expression levels [11]. Since this assumption violates equilibrium thermodynamics, our model is more suitable for describing *in vitro* immunoEM data in absence of energy sources from the cell. Note that in living cells clustering may in part be regulated by active, energy-dependent mechanisms. For instance, clustering and signaling of constitutively active K-RasG12V depends on the presence of actin fences [11]. Such membrane-associated actin filaments, part of the actin cortex, are highly dynamic and, hence, K-Ras clustering may be regulated. An expression level-independent cluster/monomer ratio has also been found for glycosyl-phosphatidylinositol-anchored proteins (GPI-AP) *in vivo* [33]. Finally, clustering of proteins in the immunological synapse is an active, actin-myosin dependent process [34], presumably to overcome the entropic barrier of localizing proteins [35].

What is the role of Ras clusters beyond simple protein sorting? Harding *et al.* argued that Ras clusters allow for highly precise coding of time-dependent inputs, termed high fidelity signaling [25], [26]. First, Ras is highly abundant in the membrane (tens of thousands molecules), hence the number of active clusters can be wide-ranging depending on input, *e.g.*, EGF. Second, clusters have a short life time (about 0.4 s). This high turnover of clusters allows for high temporal precision. Third, Ras clusters act as digital nanoswitches, which may lead to noise reduction in the signal transmission step across the membrane due to coarse graining and averaging of rapidly fluctuating signals. In fact, Ras clusters have similarity to an analog-digital-analog converter known from engineering, transmitting analog EGF input into analog ERKpp output [25], [26]. This design principle was indeed recently confirmed by experiments on the Ras-Raf-MEK-ERK pathway in baby hamster kidney cells. In particular, experiments showed that Ras clusters (actually Raf*-tH with varying kinase activity) are fully active even for small inputs (kinase activities) [27], and that the total amount of active Ras (concentration of K-Ras  GTP) and ERKpp are proportional to EGF input [24]. Such analog ERK activation was recently also observed in proliferating mammalian fibroblasts [36].

The above listed properties of Ras clusters are supported by our model. According to Fig. 7A, Ras clusters are fully active, even for small inputs. Nevertheless, the number of Ras clusters and hence the total activity of all Ras molecules in the membrane are approximately proportional to the input (Fig. 7B), allowing faithful transmission of continuous, time-dependent input signals. Interestingly, the activity of a single Ras molecule and of non-interacting Ras molecules are also approximately proportional to the input (Fig. 8A and Fig. 8B, respectively). However, signaling by Ras clusters is less noisy and, hence, Ras clusters can transmit signals more robustly than non-interacting Ras molecules without clustering ability. The activity of Ras in clusters exhibits smaller intrinsic noise from random switching between active and inactive states (Fig. 7A and B, black error bars). The activity of Ras in clusters is also less sensitive to extrinsic noise from fast fluctuations in input than non-interacting Ras molecules (by comparison of green error bars in Fig. 7A and Fig. 8A, respectively). The reason for the noise reduction by clusters is that Ras clusters are fully active, suppressing random switching between active and inactive states, as well as activity changes due to fluctuations in input.

Our model relies on the short-range attraction and long-range repulsion between Ras molecules. The physical origin of these interactions are yet to be determined. However, the attraction may originate from direct Ras-Ras interaction via hydrophobic, van der Waals, or electrostatic interactions [37], but may also be mediated indirectly by scaffold proteins and lipids [17], [23], [37]. The latter mechanism is supported by the finding that the positively-charged polybasic C-terminus of K-Ras binds negatively charged phospholipids and sequesters acidic phospholipids, which may attract even more K-Ras molecules. Furthermore, that mutant GFP-K-RasG12V S181D has a reduced ability to bind the membrane, as well as to cluster [37]. Such a lipid-mediated mechanism would support the concept of dynamic lipid rafts, which only form in presence of an activated membrane protein such as Ras  GTP. The physical origin of the repulsion is harder to pinpoint, but may result from induced membrane curvature as a result of insertion of the farnesyl-polybasic anchor of K-Ras or the farnesyl-palmitate anchor of H-Ras into the inner leaflet of the membrane [17]. Lipid-anchor induced membrane deformations are supported by molecular dynamics simulations [38], and may lead to long-range repulsion [39]. Furthermore, recent experiments explicitly show that smallGTPases (Arf) induce membrane curvature [40].

There are certain shortcomings of the immunoEM data, rendering the cluster analysis of immuno-gold data difficult. In our simulations, the addition of gold particles to completely random distributions of Ras molecules produced the following. If more than one gold-labeled antibody is allowed to bind a Ras molecule, provided there are no steric clashes between gold particles, the 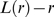 plot still predicts Ras clustering. Varying the antibody length systematically resulted in distance 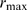 being approximately equal to the length of the antibody. This is due to a small fraction of Ras molecules being associated with multiple gold particles: these gold particles will be within two antibody lengths of each other, and approximately one antibody length from each other on average, resulting in a peak in the 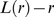 plot. This suggests that, unless it is verified that each Ras can only bind a single antibody ( *i.e.* the anti-GFP antibody can only bind a single epitope on GFP fused to a Ras molecule), immunoEM data can overestimate clustering. In contrast, two clustered Ras molecules in contact with each other could each interact with separate antigen-binding regions of the same antibody, since an antibody has two antigen-binding regions. In this case the cluster of two Ras molecules is unobservable by immunoEM, leading to an understimation of clustering in the gold point patterns. These issues would have to be addressed if immunoEM studies are to form the basis of an accurate quantification of Ras clustering.

In conclusion, a comprehensive description of Ras clustering is an essential step in the understanding of Ras signaling properties, and of small, inner-membrane GTPases in general. For instance, we showed that clustering leads to robustness to noise, especially input noise (Figs. 7 and 8). While the model we have analyzed fits the data (Figs. 2 and 7), several questions remain unanswered. For one, lack of high resolution structural information about clustered Ras molecules prevents us from “seeing” clearly into the physicochemical basis of clustering (only partial crystal structures of Ras molecules exist [41]). There is also the possibility of regulation of clusters from within utilizing specific lipids and scaffold proteins, which has scarcely been addressed in the literature thus far [17], but would provide critical details to the construction of an accurate model for clustering. While we have shown that immunoEM data can aid in the visualization of Ras clusters, data regarding the dynamics of clustering are found in the form of SPT [23], FRET [21], fluorescence recovery after photobleaching (FRAP) [42], and single-molecule fluorescence microscopy studies [22], [43]. These data remain to be integrated into more detailed spatio-temporal Monte Carlo simulations, so that the exchange of proteins between freely diffusing monomers in the membrane and immobile clusters can be investigated [44]. Interestingly, our biophysical model of Ras clustering shares the long-range repulsion due to elasticity with recent models of lipid microphase separation [39] and chemoreceptor clustering [45], [46] in bacteria. Hence, similar biophysical principles may govern the clustering of very different types of proteins in prokaryotic and eukaryotic membranes. The latter may include EGF and Fc *γ* receptors, which are believed to associate with rafts or to form small clusters [47], [48].

## Methods

### Experimental immunoEM data

Relevant experiments are described in [7], [11], [24], [27]. Briefly, Ras clustering was examined on intact 2-D sheets of apical plasma membrane, ripped off from adherent baby hamster kidney cells directly onto EM grids. Ras-fluorescent protein fusion constructs were used, including the minimal plasma membrane targeting motif (lipid anchor) of H-Ras fused to GFP (GFP-tH) or RFP (RFP-tH), as well as constitutively active H-RasG12V and K-RasG12V. These are tagged using affinity-purified polyclonal anti-GFP antibodies, conjugated with 4 nm gold particles, and visualized using electron microscopy (immunoEM). The resulting point patterns of gold particles were analysed for clustering (see below for details). Ras isoform clustering was found to depend differentially on membrane-associated actin [11], lipid-raft constituent cholesterol [7], and scaffold proteins galectin-1, galectin-3, and Sur-8 [49]. For a recent review see [18].

### Biophysical model

A Ras molecule in the membrane can be in either one of two states, active (on) with energy 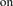 or inactive (off) with energy 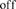 [50]. For wild-type Ras, the active (inactive) state corresponds to Ras  GTP (Ras  GDP). More generally, the two states correspond to two different protein conformations, making the two-state model applicable to activity mutants and lipid anchors as well. For any such two level system, the probability for a single Ras molecule to be active is
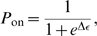
(1)where 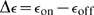 is the free-energy difference between the active and inactive states. While Eq. 1 is not explicitly used for our simulations as it describes the activity of Ras in absense of interactions, it builds intuition about parameter 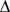. This parameter is effectively determined by the input signal of the pathway, *e.g.* EGF, except for the Ras activity mutants and lipid anchors, where it desribes an energetic bias in conformational state.

To describe clustering of active Ras molecules, as directly observed for K-Ras and H-Ras using *in vivo* FRET [21], we introduce short-range attraction *J* between active Ras molecules, driving cluster formation. In order to limit cluster size, we introduce long-range repulsion 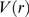, where *r* is the distance between two Ras molecules. For the repulsive interaction energy, we use a Gaussian function as previously applied for describing microphase separation of lipid mixtures [39]

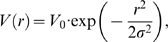
(2)where 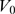 is the maximal repulsion for two Ras molecules in close proximity and 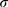 is the width, i.e. the range of the repulsion beyond which the potential drops quickly (Fig. 3A). The frustration between short-range attraction and long-range repulsion leads to small clusters. More precisely, the optimal cluster size corresponds to the minimum of the cluster energy divided by the number of Ras molecules in the cluster, i.e. the energy density [51]. The parameters used in this study are 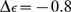, 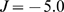, 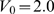, and 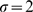
nm. Long-range repulsions were neglected beyond a 6 nm cut-off to reduce the calculation time. All energies are in thermal energy units 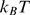 with 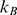 being the Boltzmann constant and *T* the absolute temperature.

### Monte Carlo simulations

Since the immunoEM data are obtained from *in vitro* membrane sheets, clustering is an equilibrium process. Models of such phenomena are therefore particularly amenable to Monte Carlo simulations, which include energetics as well as entropy [51]. To set up simulations, we discretize a defined area of the plasma membrane inner leaflet to obtain a two-dimensional 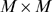 square-lattice where *M* is the lattice size. On the lattice, each position is uniquely described by an index *i* (if the 2-D lattice is thought of as a linear array of length 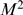). We assign a Boolean value 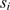 to every Ras, where 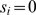 if Ras *i* is active, and 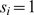 if Ras *i* is inactive. Using this notation, we can construct an energy function describing the total energy *E* for a set of *N* molecules
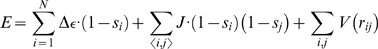
(3)where 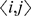 denotes nearest-neighbor pairs.

After randomly generating the positions of the starting Ras molecules on the lattice, individual Ras molecules are chosen at random and attempted to move to a new location on the lattice. Included in each step is a probability of switching between active and inactive Ras. Moves are accepted or rejected based on the Metropolis-Hastings algorithm. We use a lattice of size 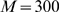 and a lattice constant 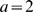
nm (the size of a Ras molecule [44]), resulting in a 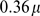
m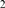 membrane. In order to reduce boundary effects of the lattice, we adopt periodic boundary conditions.

### Gold particles

In order to compare the simulation outputs with immunoEM data, gold-labeled antibodies are added to the equilibrated Ras molecules. The length of the antibody used in the experiments is 10 nm [11]. When an antibody binds a Ras molecule, the gold particle associated with the antibody can at any one time occupy any position on the surface of a hemisphere around the Ras molecule (Fig. 3B). For simplicity, the radius of the hemisphere is chosen equal to the length of the antibody, and the centre of the gold particle's position is projected onto the plane of the membrane, defining the particle's position on the lattice. This position is then matched against previous gold positions for steric clashes, and if it is found to be closer than 4 nm from another gold particle, the position is rejected and another Ras is picked at random. This process is iterated until 42% of Ras molecules are occupied, corresponding to the experimentally-observed capture ratio [11].

### Cluster analysis

We use three functions to evaluate the degree of Ras clustering: 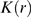, 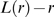 and 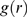. Ripley's *K*-function 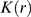 was first proposed for analyzing spatial point patterns [52]. 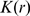 calculates the expected number of particles within a distance *r* of any particle, normalized by the average density *λ*

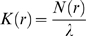
(4)

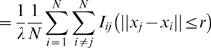
(5)

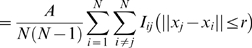
(6)where *A* is the area of the lattice studied, *N* the number of Ras molecules or gold particles, *λ* the surface density and 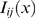 an indicator function which takes a value of 1 if 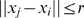 and 0 otherwise. Under the null hypothesis of complete spatial randomness, 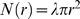, so 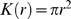. An often used non-linear transformation of 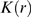 which we shall employ is [7]

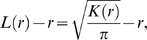
(7)which has a value of 0 for complete spatial randomness, is positive for clustering, and negative for depletion of particles. For large *r*, 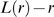 is zero on average, since particles are uncorrelated. Since 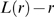 is a non-linear transformation of 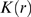, when averaging over multiple simulations to resemble a large piece of membrane (see below), the 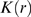 values are averaged first and only then transformed into 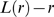. For further analyses of simulations, we use summary statistics 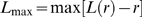 and corresponding distance 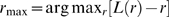.

The pair-correlation function 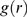 can be defined in two different ways. The first by normalizing and differentiating 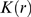 [53], [54]

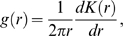
(8)and the second by counting in a similar manner to 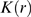 but in concentric rings
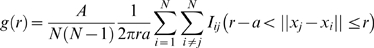
(9)for 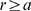, where *a* is the lattice constant. Testing these two versions of the pair-correlation function yielded slightly different absolute values of 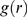, but the relative behaviors of the two were identical. For a random distribution of particles and for large *r* in general, 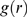 takes a value of 1 on average. We again use summary statistics 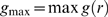 and 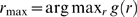.

To estimate confidence intervals for the 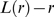 cluster analysis, 99 simulations were run for each density with all interactions set to zero, simulating a random distribution of Ras in order to obtain an estimate of the background clustering noise intrinsic to each density. Triplets of 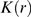 were averaged to simulate a 1 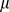
m^2^ membrane as used in experiments, and 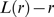 values were calculated for each. The 68.3%, 95.4%, and 99.0% confidence intervals for individual measurements were obtained by multiplying the standard deviation of the 33 triplets by 1, 2, and 2.576, respectively. Standard deviations for 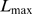, 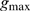, and 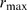 were calculated based on triplets as well.

### Accession Numbers

The primary protein accession numbers from the Swiss-Prot databank (http://www.ebi.ac.uk/swissprot/) for the proteins mentioned in the text are: H-Ras P01112, K-Ras P01116, and N-Ras P01111.
